# Correction to: Gender-responsive monitoring and evaluation for health systems

**DOI:** 10.1093/heapol/czae103

**Published:** 2024-11-11

This is a correction to: Rosemary Morgan, Anna Kalbarczyk, Michele Decker, Shatha Elnakib, Tak Igusa, Amy Luo, Ayoyemi Toheeb Oladimeji, Milly Nakatabira, David H Peters, Indira Prihartono, Anju Malhotra, Gender-responsive monitoring and evaluation for health systems, *Health Policy and Planning*, Volume 39, Issue 9, November 2024, Pages 1000–1005, https://doi.org/10.1093/heapol/czae073

In the originally published version of the manuscript, an affiliation was missing for the final author. The enumeration in the author list should read: ‘Anju Malhotra^1,**5**^’ instead of: ‘Anju Malhotra^1^’. The published list has had the additional affiliation added: ‘^5^The Global Financing Facility, The World Bank 1818 H Street NW, Washington, DC 20433’.

The emendations have been made to the article.

